# Pregnancy rates and outcomes in a longitudinal HIV cohort in the context of evolving antiretroviral treatment provision in South Africa

**DOI:** 10.1186/s12884-022-04829-2

**Published:** 2022-07-26

**Authors:** Nivashnee Naicker, Nonhlanhla Yende-Zuma, Ayesha B. M. Kharsany, Hlengiwe Shozi, Duduzile Nkosi, Anushka Naidoo, Nigel Garrett, Salim S. Abdool Karim

**Affiliations:** 1 Centre for the AIDS Programme of Research in South Africa (CAPRISA), Doris Duke Medical Research Institute (2nd floor), Nelson R Mandela School of Medicine, University of KwaZulu–Natal, Private Bag X7, Congella, Durban, 4013 South Africa; 2 School of Laboratory Medicine and Medical Science, Nelson R. Mandela School of Medicine, University of KwaZulu-Natal, Durban, South Africa; 3 Department of Public Health Medicine, School of Nursing and Public Health, University of KwaZulu-Natal, Durban, South Africa; 4 Department of Epidemiology, Mailman School of Public Health, Columbia University, New York, New York NY 10032 USA

**Keywords:** Pregnancy, HIV infection, Antiretroviral therapy

## Abstract

**Background:**

In South Africa, women continue to face a high burden of Human Immunodeficiency Virus (HIV) infection and the possible complications thereof during pregnancy. We assessed pregnancy incidence rates and outcomes in a longitudinal HIV cohort study over a 15-year period.

**Methods:**

We evaluated pregnancies among women ≥ 18 years between 2004 and 2019 in the CAPRISA 002 study. We analysed pregnancy rates following HIV acquisition, CD4 counts and HIV viral load dynamics and pregnancy outcomes. We used linear regression to assess if the mean CD4 and log_10_ viral load close to delivery increases or decreases linearly across three different timepoints.

**Results:**

In total 245 women enrolled into the HIV negative study phase, 225 into the HIV infection phase and 232 in the antiretroviral therapy (ART) phase. Median follow-up time was 2.0 years [Interquartile Range (IQR) 0.8–2.0] during the HIV negative phase, 2.6 years; (IQR) 1.2–4.8] during HIV infection and 3.7 years (IQR 1.8–5.0) on ART, with maximum follow-up time of 2, 10 and 6 years respectively. Overall, 169 pregnancies occurred in 140 women, of which 16 pregnancies were observed during acute or early HIV infection [Incidence Rate (IR) 8.0 per 100 women-years; 95% confidence interval (CI): 4.6—12.9], 48 during established infection [IR 9.3; (CI 6.8–12.3)] and 68 on ART [IR 8.9; (CI: 7.0 – 11.4)]. Birth outcomes from 155/169 (91.7%) pregnancies were 118 (76.1%) full term live births, 17 (10.9%) premature live births, 9 (5.8%) therapeutic/elective miscarriages, 8 (5.1%) spontaneous miscarriages and 3 (1.9%) spontaneous foetal deaths or stillbirths. Six mother-to-child transmission events occurred, with four documented prior to 2008. Over time, mean CD4 count in pregnant women increased from 395 cells/µL (2004—2009) to 543 cells/µL (2010–2014) and to 696 cells/µL (2015–2019), *p* < 0.001. Conversely, the viral load declined from 4.2 log_10_ copies/ml to 2.5 log_10_ copies/ml and to 1.2 log_10_ copies/ml (*p* < 0.001) for the corresponding periods.

**Conclusions:**

Pregnancy rates following HIV acquisition were high, emphasising a need for timeous ART provision and contraception counselling in women recently diagnosed with HIV. CD4 count and HIV viral load trajectories reflect improvements in treatment guidance for pregnant women over time.

## Background

In South Africa, young women bear the dual burden of high rates of Human Immunodeficiency Virus (HIV) infection and unintended pregnancies, consequently bearing high rates of HIV associated pregnancy complications [1]. These may include direct obstetric complications such as haemorrhage or sepsis and non-pregnancy related infections including tuberculosis and meningitis [1, 2]. Among pregnant women attending public sector facilities in 2019, the national HIV prevalence was 30%, with the highest proportion of 40.9% among pregnant women in the province of KwaZulu-Natal [3]. With high rates of unplanned pregnancies [4, 5], it is not known whether HIV acquisition and subsequent antiretroviral therapy (ART) initiation impacts on pregnancy incidence, as well as pregnancy outcomes in this setting.

As a consequence of improved and rapidly evolving treatment guidance for pregnant women and improved access to ART and safer medication [6], there has been a substantial decline in HIV related maternal mortality rates [7]. Pregnant women are prioritised for rapid HIV testing to improve knowledge of HIV positive status, initiation and continuation of combination ART as early as possible in pregnancy [8]. Single-drug prevention of mother to child transmission (PMTCT) prophylaxis, used early in the epidemic, has almost completely been replaced by combination ART for treatment of HIV infection and PMTCT in pregnant women. Early ART provision in pregnancy has led to a concomitant decline in mother-to-child transmission events from 8% in 2008 to 3.5% in 2010 and 0.9% in 2017 [9]. While ART has had a positive impact on maternal health and in reducing HIV transmission, ART use in pregnancy may be associated with adverse birth outcomes, including preterm delivery and low birth weight, which is more likely to occur in women who initiate ART prior to conception [10–12].

Nonetheless, improved maternal outcomes for HIV positive pregnant women in the era of ART has impacted pregnancy desires [13–15]. Studies from Africa have shown that pregnancy desire among women initiated on ART was substantial, at around 40% and was associated with older age and lower parity [13, 14]. In Johannesburg, South Africa, high rates of unplanned pregnancies were found to be common in women receiving ART, irrespective of time on ART, with an associated high rate of pregnancy loss [15]. However, in KwaZulu, Natal, South Africa, the epicentre of the HIV epidemic, there is limited data on pregnancy rates among women soon after HIV acquisition and following ART initiation.

The aim of this study was to assess pregnancy incidence rates and pregnancy outcomes, after HIV acquisition and on ART in women enrolled in the CAPRISA 002 study.

## Methods

### Study setting and study population

**Fig. 1:**
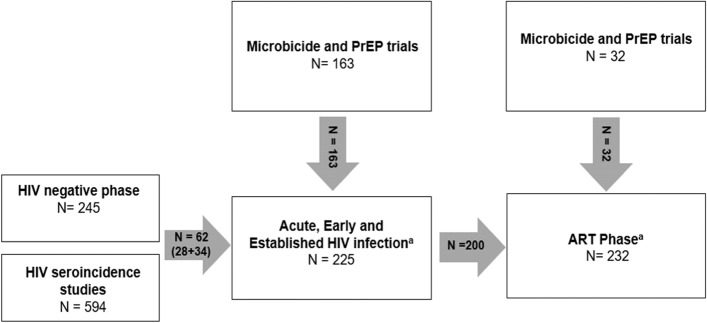
Enrolment of participants into the CAPRISA 002 Study (2004 – 2019) ^a^ 33 women initiated ART in the CAPRISA 009 study and re-entered the ART phase

The Centre for the AIDS Programme of Research in South Africa (CAPRISA) 002 study is a prospective cohort study which aimed to advance the understanding of HIV-1 subtype C acquisition, pathogenesis and clinical disease progression. The study was initiated in August 2004 and HIV negative women at high risk of HIV acquisition were enrolled at an urban clinical research site in eThekwini, Durban and a rural site in Vulindlela, KwaZulu-Natal, South Africa. The study recruitment procedures and eligibility criteria have been described previously [16]. Briefly, women 18 years and older self-identifying as sex workers or having had at least three partners in the 3 months prior to recruitment, and testing HIV negative were eligible for study participation [17]. Pregnant women were not eligible for enrolment into the HIV negative phase of the study due to the short follow-up period and nature of employment [16]. In the HIV negative study phase, women were followed up monthly for a maximum of 24 months or until diagnosis of HIV infection, at which time they were offered enrolment into the acute HIV infection phase of the study and followed-up during established HIV infection until they were ART requiring [16]. Acute HIV infection is defined as 0–3 months post-diagnosis of HIV infection, early HIV infection as ≥ 3–12 months post-diagnosis of HIV infection and established HIV infection as > 12 months post-HIV diagnosis, as per study protocol. In addition, CAPRISA undertakes observational studies in preparation for clinical trials in HIV prevention including microbicide and pre-exposure prophylaxis trials [18, 19]. Women acquiring HIV in any of these studies were also offered enrolment and long-term follow-up in the CAPRISA 002 study (Fig. 1). HIV infection as endpoint was based on a positive HIV-1 antibody test with a previously documented negative HIV-1 antibody test within 5 months; or the presence of HIV-1 RNA in the absence of HIV antibodies [16, 17]. Estimated time of HIV infection was defined as the midpoint between the last negative HIV ELISA and the first positive HIV ELISA or 14 days prior to a positive HIV-1 RNA test if the HIV ELISA is negative on the same day [16].

Once initiated on ART, participants have been in follow-up for up to 6 years [median 3.7 years, Interquartile range (IQR) 1.8–5.0] and remain in follow-up [20]. A subset of women were initiated on treatment in the CAPRISA 009 study, a randomised controlled trial which assessed ART outcomes in women exposed to tenofovir gel, when eligible for ART [21]. These women were transitioned back into the treatment phase of the CAPRISA 002 study once their participation in the CAPRISA 009 study ended.

### Clinical evaluation

All participants provided written informed consent upon entry into the CAPRISA 002 study. Participants underwent clinical evaluation and had peripheral blood and urine samples collected at each scheduled study visit. Pregnancy status was determined by on-site urine pregnancy testing (Quickview One-Step hCG Combo Test, Quidel Corporation, San Diego, CA, USA) with minimum detection limit of 25mIU/mL or other standard urine pregnancy test, as available. Pregnancy testing was performed by trained nurses at entry into each study phase and on clinical suspicion of pregnancy at any study visit [16]. Estimated dates of conception, gestational age and pregnancy delivery were calculated from the date of last menstrual period. As standard of care all participants were offered contraception counselling and provision during the study. Participants who became pregnant were referred for antenatal care through the preferred public or private sector primary health care provider.

Based on the contemporaneous South African national treatment guidelines, ART was initiated at a CD4 count eligibility threshold applicable at the time or the presence of an AIDS-defining illness or pregnancy [20]. Pregnant women, if not eligible for combination ART at the time of their pregnancy, were offered a PMTCT single or dual drug regimen as standard of care. Prior to 2010 in South Africa, the ART initiation criterion was CD4 count threshold < 200 cells/µl or World Health Organisation (WHO) stage 4 and single dose nevirapine prophylaxis for women with a CD4 count above this threshold [6]. In 2010, WHO Option A was introduced, lowering the ART initiation threshold to CD4 count of < 350 cells/µl and adding zidovudine PMTCT prophylaxis from 14 weeks of gestation [6]. In 2013, Option B was implemented which included Option A but added pregnant women to initiate ART irrespective of CD4 count, to continue with ART throughout pregnancy and delivery until one-week post cessation of breastfeeding [6]. In 2015, Option B + was introduced which recommended the initiation of ART at any CD4 count and more importantly to continue on lifelong ART [6]. Pregnant women in the CAPRISA 009 study (2011 – 2014) were initiated on ART irrespective of CD4 count. Combination ART was provided to all pregnant women at the research clinic, while PMTCT prophylaxis regimens were accessed through the antenatal care facilities. In this analysis viral suppression is defined as HIV viral load < 400 copies/ml.

### Laboratory evaluation

HIV infection was diagnosed using rapid tests (Determine: Abbott Laboratories, Tokyo, Japan and Capillus; Trinity Biotech, Jamestown, NY, USA) with confirmatory HIV enzyme immunoassay (BEP 2000; Dade Behring, Marburg, Germany) used for discordant antibody tests [16]. All participants had full haematological assessments including CD4 count (FACSCalibur Flow Cytometer or TruCOUNT, BD biosciences, San Jose, CA, USA) and HIV viral load (Roche Cobas Amplicor v.1.5, Taqman version 1.0 or version 2.0, Roche Diagnostics, Basel, Switzerland) monitoring was done at three to six-monthly intervals. STI screening, including testing for *Neisseria gonorrhoeae*, *Chlamydia trachomatis*, *Trichomonas vaginalis*, *Mycoplasma genitalium*, Herpes Simplex Virus (HSV) type 2 and bacterial vaginosis, was performed at the same frequency as previously described [22]. Testing for syphilis was provided by the primary health care provider.

### Data management

All participant clinical, on-site testing and laboratory results were documented in study source notes and or case report form (CRF) by the study nurse or study clinician. A Pregnancy Outcome CRF was completed following face to face interview with a participant once a pregnancy outcome was reached. Pregnancy and laboratory data were captured onto standardised CRFs identifiable only by a participant identification number to maintain participant confidentiality. CRFs were faxed using the DataFax system (Clinical DataFax Sytems Inc., Ontario, Canada), with all data verified by data encoders for quality checks and stored in a secure study specific database.

### Statistical analysis

Baseline and follow-up characteristics were summarized as either medians with interquartile range or means with standard deviation for continuous variables and proportions for categorical variables. Pregnancy incidence rates were calculated for the HIV negative study phase, acute, early and established HIV infection and post ART initiation separately. In women with more than one pregnancy in the same study phase, only the first pregnancy was included in the incidence calculation and Kaplan–Meier curve. However, if the same woman had pregnancies in different phases, all pregnancies were included in the survival analyses. For pregnancy outcomes, all pregnancies were counted including repeat pregnancies in each study phase.

Prior to HIV seroconversion, pregnancy-free survival time was calculated from enrollment to time of conception, last visit date for those who were terminated early without seroconverting, last date prior to estimated date of HIV infection (for those who seroconverted) or at 24 months post enrollment for those who completed the HIV negative phase without seroconverting. Whereas, after HIV acquisition, pregnancy-free survival time was calculated from estimated date of HIV infection until either time of conception or last visit date prior to ART initiation. Moreover, in the ART phase it was calculated from ART initiation date to time of conception, last visit date prior to or at 5-year post ART initiation (administrative censoring period). We used Poisson approximations to calculate 95% confidence interval (CI) for pregnancy incidence rates. Kaplan–Meier cumulative probability of falling pregnant was calculated separately for each phase. CD4 count and HIV viral load measurements close to the time of the delivery were compared over time using linear regression to assess for linear trend.

### Ethics approval and consent to participate

The CAPRISA 002 study was reviewed and approved by the University of KwaZulu-Natal Biomedical Research Ethics Committee (E013/04). All methods were carried out in accordance with relevant guidelines and regulations. All study participants provided written informed consent at study entry.

## Results

### Baseline characteristics

A total of 245 women were enrolled into the HIV negative study phase, 225 women into the acute to chronic HIV infection study phase and 232 women into the ART phase (Fig. 1). Median follow-up time was 2.0 years; [Interquartile Range (IQR) 0.8–2.0] during the HIV negative phase, 2.6 years; IQR (1.2–4.8) years during HIV infection and 3.7 years, IQR (1.8–5.0) on ART with a maximum follow-up period of 2 years, 10 years and 6 years respectively. Women became pregnant at a median age of 23 (range 20.5–26.5) years in the HIV negative phase, 26.5 (range 24–30) years in the HIV infection phase and 31 (range 28–33) years in the treatment phase respectively (Table 1). The majority of pregnant women reported being in a stable partnership pre- (71.9%) and post-ART initiation (79.4%), but very few were married (1.6% and 2.9%, respectively). A large proportion of women (60%), who acquired HIV and were ART naive reported having had at least one previous pregnancy.

**Table 1:** Baseline socio-demographic and clinical characteristics of pregnant and non-pregnant women enrolled in the CAPRISA 002 Study, KwaZulu-Natal Province (2004–2019)

	**HIV negative**		**HIV infection (Acute, Early, Established infection), ART naive**		**HIV infection on ART**	
	**Pregnant** **(** ***N*** ** = 24)**	**Non-Pregnant** **(** ***N*** ** = 224)**	**Pregnant** **(** ***N*** ** = 64)**	**Non-pregnant** **(** ***N*** ** = 161)**	**Pregnant** **(** ***N*** ** = 68)**	**Non-pregnant** **(** ***N*** ** = 164)**
***Socio-demographic***						
**Median age at enrollment (IQR), years**	22.0 (19.0—26.0)	37.5 (26.0—43.0)	24.0 (22.0—27.5)	25.0 (22.0—31.0)	29.0 (26.0—32.0)	29.0 (26.0—34.0)
**Median age at pregnancy (IQR), years**	23 (20.5–26.5)		26.5 (24–30)		31 (28–33)	
**Age group, years (n, %)**						
18–24	14 (58.3)	32 (14.3)	28 (43.8)	57 (35.4)	6 (8.8)	16 (9.8)
> 24	10 (41.7)	192 (85.7)	36 (56.3)	104 (64.6)	62 (91.2)	148 (90.2)
**Highest level of education (n, %)**^a^						
< grade 8	0	60 (26.8)	2 (3.1)	9 (5.6)		10 (6.1)
≥ grade 8	24 (100.0)	164 (73.2)	62 (96.9)	152 (94.4)	68 (100.0)	154 (93.9)
**Relationship status (n, %)**						
Married	0	16 (7.1)	1 (1.6)	8 (5.0)	2 (2.9)	7 (4.3)
Stable partner	9 (37.5)	64 (28.6)	46 (71.9)	114 (70.8)	54 (79.4)	116 (70.7)
Casual partner(s)	12 (50.0)	127 (56.7)	7 (10.9)	15 (9.3)	1 (1.5)	18 (11.0)
Other^**b**^	3 (12.5)	17 (7.6)	10 (15.6)	24 (14.9)	11 (16.2)	23 (14.0)
**Number of dependents (n, %)**						
0–1	19 (79.2)	75 (33.6)	47 (74.6)	98 (62.0)	45 (68.2)	105 (64.0)
> 1	5 (20.8)	148 (66.4)	16 (25.4)	60 (38.0)	21 (31.8)	59 (36.0)
**Pregnant before (n, %)**						
Yes	2 (33.3)		36 (60.0)		51 (76.1)	
No	4 (66.7)		24 (40.0)		16 (23.9)	
***Laboratory evaluations at enrolment into each study phase***						
Median CD4 count (IQR), cell/µL	-	-	519 (417—696)	526 (420—667)	423 (313—601)	413 (287—566)
Mean log10viral load (SD), copies/ml	-	-	4.3 (0.8)	4.5 (0.9)	4.2 (1.0)	4.0 (1.1)

^a^ missing data

^b^ combination of single, divorced, widowed, separated, refused to answer

### Pregnancy incidence and timing of ART initiation

During the study period, 140 women had 169 pregnancies, of which 156 were the first pregnancies in each study phase. A single pregnancy was observed in 113 women while 25 women became pregnant twice during study follow-up and two women had three on-study pregnancies. In women who were HIV negative, 24 pregnancies were observed [Incidence Rate (IR) 6.2 per 100 women years (wy); 95% confidence interval (CI): 4.0 – 9.3] and 64 pregnancies occurred in women with HIV infection, but not on ART [IR 8.9 per 100 wy; 95%CI: 6.9—11.4] (Table 2). Among these, the pregnancy incidence rate in acute and early HIV infection was 8.0 per 100 wy (95%CI: 4.6—12.9) and 9.3 per 100 wy (95%CI: 6.8 – 12.3) among those with established infection. In women stable on ART, 68 pregnancies occurred (IR 8.9 per 100 wy; 95%CI: 7.0—11.4).

**Table 2:** Pregnancy incidence by HIV and ART status

	**Number of women**	**Number of women-years**	**Number of pregnancies**	**Pregnancy incidence rate per 100 women-years (95% CI)**
HIV negative	248	385.0	24	6.2 (4.0 – 9.3)
HIV infection, ART naïve	225	718.6	64	8.9 (6.9 – 11.4)
• Acute/Early HIV infection	225	200.8	16	8.0 (4.6 – 12.9)
• Established HIV infection	174	517.8	48	9.3 (6.8–12.3)
HIV infection on ART	232	758.7	68	8.9 (7.0 – 11.4)

Of the women who were ART naïve when they became pregnant, 36/70 (51.4%) initiated a triple ART regimen during pregnancy, 23/70 (32.9%) received only PMTCT prophylaxis or did not receive ART nor PMTCT prophylaxis 11/70 (15.7%) [Table 3]. Of women who had viral load monitoring close to the time of delivery and who initiated ART during pregnancy, half 30/59 (50.8%) did not achieve virological suppression prior to delivery (Table 3). In contrast, the majority of women 45/55 (81.8%) who became pregnant after ART initiation, were virologically suppressed prior to delivery.

**Table 3:** Pregnancy outcomes of women enrolled in the CAPRISA 002 Study, KwaZulu-Natal Province (2004–2019)^a^

	**HIV negative** **(** ***N*** ** = 25 pregnancies –21 with known outcomes)**^**b**^	**HIV infection, ART naïve** **(** ***N*** ** = 70 pregnancies –68 with known outcomes) **^**c,d**^	**HIV infection on ART** **(** ***N*** ** = 74 –66 with known outcomes) **^**e,f**^
**Full term live birth > 37 weeks** **(n, %)**	15 (71.4)	53 (77.9)	50 (75.8)
*Delivery method*	*NVD* = *2 (13.3)*	*NVD* = *30 (56.6)*	*NVD* = *28 (56.0)*
	*C-section* = *3 (20)*	*C-section* = *21 (39.6)*	*C-section* = *20 (40.0)*
	*Unknown* = *10 (66.7)*	*Unknown* = *2 (3.8)*	*Unknown* = *2(4.0)*
**Premature live birth < 37 weeks** **(n, %)**	4 (19.0)	6 (8.8)	7 (10.6)
*Delivery method*	*Unknown* = *4*	*NVD* = *3* *C-section* = *3*	*NVD* = *4* *C-section* = *3*
**Spontaneous fetal death and/or still birth > 20 weeks (n, %)**	1 (4.8)	1 (1.4)	1 (1.5)
**Spontaneous miscarriage < 20 weeks (n, %)**	0	4 (5.9)	4 (6.1)
**Therapeutic/elective miscarriage (n, %)**	1 (4.8)	4 (5.9)	4 (6.1)
**Other adverse birth outcomes**^**g**^	0	4 (5.9)	6 (9.1)
***ART in pregnancy***			
MTCT prophylaxis (single or dual drug regimens) (n, %)	3 (12.0)	23 (32.9)	0
ART (n, %)	1 (4.0)	36 (51.4)	74 (100.0)
None (n, %)	21 (84.0)	11 (15.7)	0
Median months on ART prior to delivery (IQR)	**-**	5 (4—6)^h^	26 (16.5 – 47.0)^i^
Median months on ART prior to falling pregnant (IQR)	**-**	**-**	22 (12.0—40.0)
***HIV mother-to-child transmission events***			
	-	4 (5.9)	2 (3.0)
***Laboratory evaluations closer to delivery***			
Median CD4 prior to delivery (IQR), cell/µL	**-**	534 (407—652)	616 (450—753)
Detectable HIV VL (n, %)	**-**	30 (50.8)	10 (18.2)
Undetectable HIV VL (<400 copies/ml) (n, %)	**-**	29 (49.2)	45 (81.8)
Mean haemoglobin prior to delivery (SD), g/dl	**-**	11.0 (1.2)	11.7 (1.6)
***Bacterial vaginosis and STIs diagnosed before or during pregnancy in women with a full-term live birth or a premature live birth outcome***^***j***^*** (n, %)***			
Bacterial vaginosis	16 (84.2)	47 (87.0)	47 (88.7)
* Chlamydia trachomatis*	7 (36.8)	18 (33.3)	23 (43.4)
Herpes Simplex Virus (HSV) type 2	2 (10.5)	18 (33.3)	9 (17.0)
*Mycoplasma genitalium*	2 (10.5)	22 (40.7)	19 (35.8)
*Neisseria gonorrhoeae*	4 (21.1)	9 (16.7)	12 (22.6)
*Trichomonas vaginalis*	7 (36.8)	24 (44.4)	19 (35.8)

^a^ Includes 13 repeat pregnancies in each study phase

^b^ 4 outcome unknown

^c^ 2 outcome unknown

^d^ missing HIV VL in 9 participants

^e^ 5 still pregnant and 3 reached end of study before delivery

^f^ missing HIV VL in 11 participants

^g^ includes 7 early neonatal deaths, 1 low birth weight, 1 meconium aspiration,1 breech presentation with shoulder injury

^h^ for 33 women who delivered after ART initiation

^i^ for 61 women with outcomes

^j^ only the first pregnancy in each phase was considered. Out of all the 156 first pregnancies in each study phase combined, 126 full-term and pre-term birth outcomes were recorded. Therefore, denominators used were *N* = 19, *N* = 54 and *N* = 53

### Pregnancy outcomes

Pregnancy outcomes were recorded in 155 pregnancies. Of these, there were 118 (76.1%) full term live births,17 (10.9%) premature live births and 9 (5.8%) therapeutic/elective miscarriages observed (Table 3). Adverse birth outcomes, other than preterm delivery or stillbirth, were identified in ten cases: seven early neonatal deaths, one shoulder injury due to breech presentation, one meconium aspiration, and one low birth weight. Three women had adverse birth outcomes in two consecutive pregnancies. Four adverse birth outcomes were associated with a preterm delivery and two with an HIV transmission event. No congenital anomalies were identified in live births where the pregnancy outcome was known.

### HIV transmission events

Six mother-to-child transmission events were documented in the study, with four events occurring in two women in two consecutive on-study pregnancies (Table 3). All four babies tested positive at birth between 2006 and 2008. Of the other two transmission events, one infant tested positive six weeks after Caesarean section delivery in 2012, was breastfed and received six weeks of nevirapine (NVP) prophylaxis. The other infant tested positive in 2015, 10 months after delivery by Caesarean section, although there was no confirmed history of breastfeeding in this case. In both cases, the maternal viral load was detectable during early pregnancy but viral suppression was achieved prior to delivery.

### CD4 count and viral load trajectories over time

**Fig. 2:**
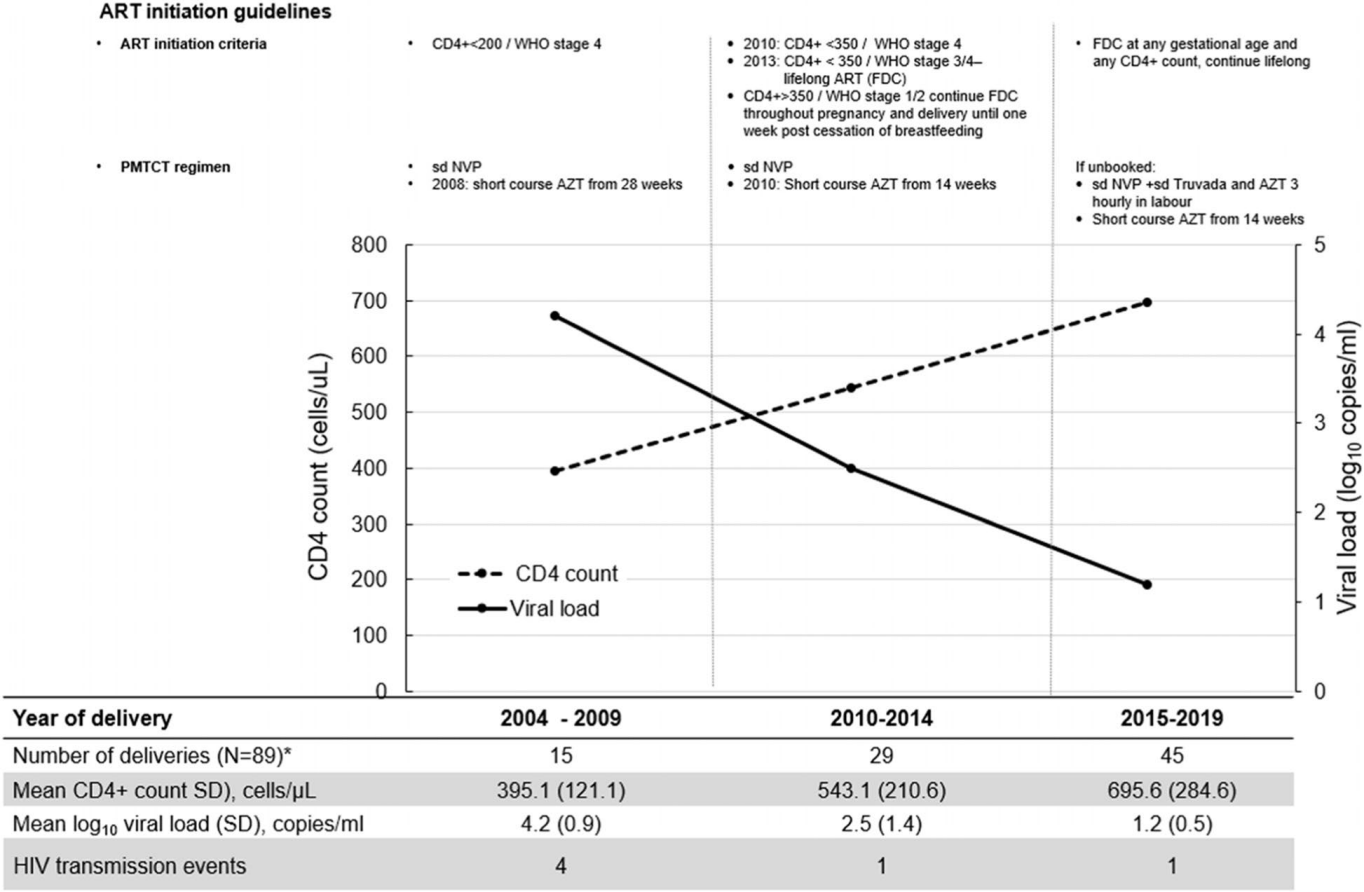
CD4 count and viral load trajectory during changing antiretroviral treatment and MTCT guidelines for pregnant women [*N* = 70] ^*^ Includes pregnancies with a full term or pre-term delivery outcome (with CD4 and viral load measured) and repeat pregnancies in the same study phase. Excludes pregnancies which occurred during the HIV negative phase and in the CAPRISA 009 study

The CD4 count and viral load were measured during pregnancy for the periods during which different ART initiation criteria applied (Fig. 2). Over time, the mean CD4 count in pregnant women increased from 395 cells/µL (2004—2009) to 543 cells/µL (2010–2014) and to 696 cells/µL (2015–2019) (test of trend *p* < 0.001). Conversely, the viral load declined from 4.2 log_10_ copies/ml to 2.5 log_10_ copies/ml and to 1.2 log_10_ copies/ml (test of trend *p* < 0.001) for each of the corresponding time periods.

### High burden of bacterial vaginosis and Sexually Transmitted Infections (STIs)

High rates of clinical and sub-clinical bacterial vaginosis and STIs including *Chlamydia trachomatis* and *Trichomonas vaginalis*, confirmed by laboratory diagnosis, were observed during or prior to pregnancy in women with a full term or preterm birth outcome (Table 3).

## Discussion

**Fig. 3:**
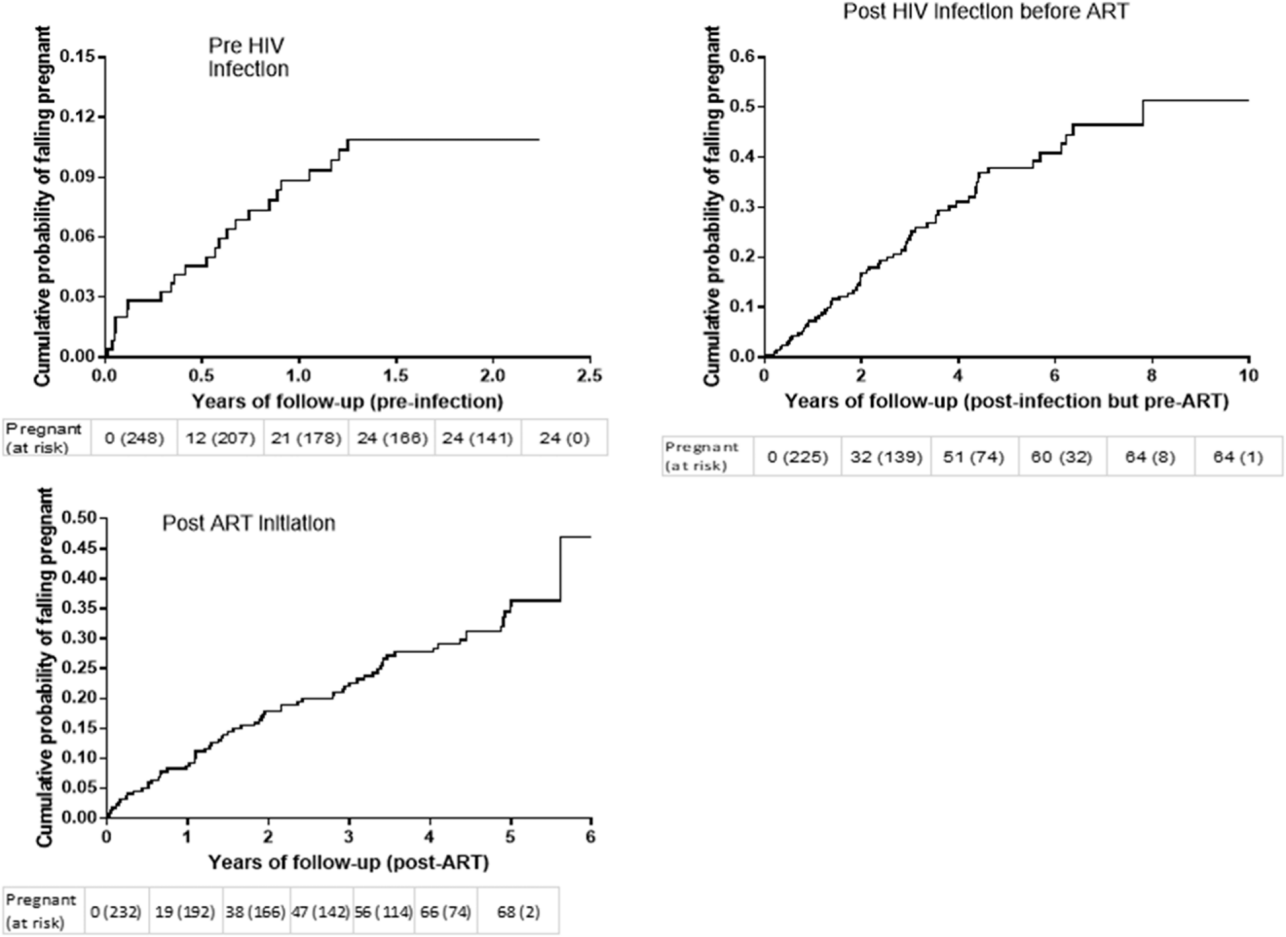
Kaplan–Meier estimates of cumulative probability of falling pregnant

In this longitudinal cohort with more than 15 years of follow-up we report pregnancy rates and associated outcomes in women before HIV acquisition, following HIV acquisition and after ART initiation. Pregnancy rates were high, even in the first year (acute and early infection) after HIV acquisition, when many women had not started ART according to the contemporaneous national guidelines (Fig. 3). Several studies, undertaken in the same region and across Africa, demonstrate similarly high pregnancy rates in women living with HIV infection and in those receiving ART [15, 23–26]. Studies evaluating pregnancy desire in women living with HIV, with or without ART, in the same region, support these findings [13, 14, 27].

Despite the provision of intensive risk reduction counselling on safer sex practices, accessibility to contraception and the adjustment to a recent diagnosis of HIV, planned or unplanned pregnancies were frequent following HIV acquisition. Furthermore, 60% of women who became pregnant after acquiring HIV reported having already had at least one previous pregnancy. Given the high levels of circulating virus during acute HIV infection, which is an independent risk factor for mother-to-child transmission [28], early initiation of ART, as recommended by current treatment guidelines, is critical to reduce vertical transmission during this period. During earlier years in this cohort, access to combination ART was not readily available to women, which is reflected in our cohort where only half of all HIV positive, ART-naïve women found to be pregnant, were started on ART before delivery.

In women stable on ART, we report a pregnancy incidence of 8.7 per 100 wy. These findings are in keeping with other studies in this setting which show that pregnancy incidence after ART initiation is substantial especially among younger women and is often unplanned [15, 23]. In our experience, pregnancies occurring during the treatment phase were more likely to be planned as most women, stable on ART, wished to complete their families and those previously experiencing fertility problems eventually conceived. The majority of women on ART achieved virological suppression prior to delivery, however it is concerning that almost 20% of women did not achieve virological suppression at the time of labour and delivery. This is in keeping with recent cohort study from South Africa in which only 63% of pregnant women were virologically suppressed at delivery [29]. In the two cases of mother-to-child transmission events which occurred in women receiving combination ART, both had documented viral loads below detectable levels prior to delivery, indicating that transmission likely occurred post-partum, or possibly early in utero.

Since PMTCT prophylaxis first became available in South Africa in 2002, treatment regimens for pregnant women, have been continuously modified and these improvements in the management of pregnant women are reflected in the substantial reduction in HIV transmission events reported [6, 9, 30]. It is reassuring that we are able to demonstrate in our cohort, a steady increase in CD4 count and a substantial reduction in viral load over time. A turning point was seen in 2015, when pregnant women were offered combination ART at any CD4 count threshold which led to the lowest mean viral load seen during the 15-year study period, translating to reduced risk of vertical transmission to unborn infants and improvement in maternal health. Although only six mother-to-child transmission events were identified in this study, this is likely to be an underestimate due to a lack of long-term follow-up data after delivery. Four babies tested positive for HIV at birth, which suggests exposure in utero, emphasising the need for early initiation of combination ART in pregnancy, which was not previously available to women. Recurrent vertical transmission in two women suggests that these women might have benefitted from preconception counselling to possibly avoid in utero transmission in subsequent pregnancies.

Pregnancy outcomes irrespective of maternal HIV and ART status were found to be similar although we are limited by the small sample size. Despite the high burden of bacterial vaginosis and STIs observed in this cohort, the rates of preterm deliveries seen is similar to background prevalence data [31]. While combination ART has been implicated in contributing to adverse pregnancy outcomes including preterm deliveries, our findings indicate a similar proportion of preterm deliveries during the HIV negative and ART phases in this cohort.

A major strength of the study was the cohort design with a 15 year follow up period and high retention rate of 95%. Limitations included incomplete data particularly for pregnancies which occurred during the HIV negative phase of the study and for long-term follow-up of mother-to-child transmission events. As this study initially enrolled sex-workers and women at high risk of HIV infection, findings of this study may be less generalisable. These women may have faced additional challenges such as poor access to health care including contraception, termination of pregnancy and ART services, and possibly had higher rates of unplanned pregnancies. Furthermore, participants received different PMTCT and ART regimens over time, in accordance with changing guidelines for pregnant women, making the data less comparable.

## Conclusions

Our study demonstrates that pregnancies occur frequently in women who have recently acquired HIV. It is therefore critical to identify and prioritise women newly diagnosed with HIV for early treatment initiation. Pregnancy outcomes were found to be similar irrespective of maternal HIV or ART status. Furthermore, this cohort provides an important overview of how the evolving treatment guidelines have positively impacted immunological and virological responses in pregnant women over time, reflecting better maternal health during pregnancy and potential reductions in mother-to-child transmission.

## Data Availability

The datasets used and/or analysed during the current study are available from the corresponding author on reasonable request.

## Abbreviations

AIDS: Acquired Immune Deficiency Syndrome
ART: Antiretroviral therapy
CAPRISA: Centre for the AIDS Programme of Research in South Africa
CRF: Case report form
HIV: Human Immunodeficiency Virus
PMTCT: Prevention of mother to child transmission
STI: Sexually Transmitted Infection
